# Crystallization kinetics of nanoconfined GeTe slabs in GeTe/TiTe_2_-like superlattices for phase change memories

**DOI:** 10.1038/s41598-024-53192-z

**Published:** 2024-02-08

**Authors:** Debdipto Acharya, Omar Abou El Kheir, Davide Campi, Marco Bernasconi

**Affiliations:** Department of Materials Science, University of Milano-Bicocca, Via R. Cozzi 55, 20125 Milan, Italy

**Keywords:** Drug development, Epidemiology

## Abstract

Superlattices made of alternating blocks of the phase change compound Sb_2_Te_3_ and of TiTe_2_ confining layers have been recently proposed for applications in neuromorphic devices. The Sb_2_Te_3_/TiTe_2_ heterostructure allows for a better control of multiple intermediate resistance states and for a lower drift with time of the electrical resistance of the amorphous phase. However, Sb_2_Te_3_ suffers from a low data retention due to a low crystallization temperature T_x_. Substituting Sb_2_Te_3_ with a phase change compound with a higher T_x_, such as GeTe, seems an interesting option in this respect. Nanoconfinement might, however, alters the crystallization kinetics with respect to the bulk. In this work, we investigated the crystallization process of GeTe nanoconfined in geometries mimicking GeTe/TiTe_2_ superlattices by means of molecular dynamics simulations with a machine learning potential. The simulations reveal that nanoconfinement induces a mild reduction in the crystal growth velocities which would not hinder the application of GeTe/TiTe_2_ heterostructures in neuromorphic devices with superior data retention.

## Introduction

Modern computing systems are based on the von Neumann architecture in which data are constantly transferred between physically separated processing and memory units. The latency and energy cost associated with shuttling data to and from the memory is a key performance bottleneck which is becoming particularly severe for the workloads of applications in artificial intelligence. Neuromorphic computing, based on artificial neurons and synapses that serve as both computing and storage units, has been proposed as an alternate approach to overcome this limitation^1^.

Resistance-based memories, such as phase-change memories (PCM) are the most promising candidates for the realization of neuromorphic devices^2^. A PCM is essentially a resistor made of a thin film of Ge_2_Sb_2_Te_5_ (GST), with an electrical resistance that differs by three orders of magnitude between the crystalline and amorphous phases^3,4^. The two states of GST can then encode a binary information which is read out in the memory by a measurement of the resistance at low bias. Joule heating induces the transformations, either amorphization via crystal melting (reset operation) or the recrystallization of the amorphous phase (set operation). The transformation is very fast (2–100 ns) and highly reversible providing cyclability in excess of 10^12^ in particular architectures^5^. Moreover, partially recrystallized intermediate states can be obtained by tuning the current pulses which can then provide intermediate values of the resistance to encode a multibit or analogic information. All these features make PCMs suitable for applications in in-memory computing devices^2^. There are, however, some serious drawbacks in the exploitation of PCMs for neuromorphic computing consisting of a cell-to-cell variability and the drift of the electrical resistance with time in the amorphous phase. The cell variability originates from the electromigration during cell programming and from the stochasticity of the crystal nucleation process^5^. The drift of the resistance is due instead to the structural relaxations, i.e. aging, of the amorphous phase^6^.

Recently, a novel type of PCMs was proposed to mitigate these problems for applications in neuromorphic computing^7^. The device consists of a superlattice made of alternating slabs of the phase change material Sb_2_Te_3_ and of a confinement slab of TiTe_2_ which acts as a thermal and diffusion barrier. TiTe_2_ always keeps the crystalline form during cycling because of its high melting temperature while Sb_2_Te_3_ undergoes the phase change. The progressive amorphization or recrystallization of several Sb_2_Te_3_ slabs enables the fabrication of a multilevel cell with several resistance states, necessary for applications in neuromorphic computing. Moreover, it was shown that the nanoconfinement of Sb_2_Te_3_ reduces the structural relaxations responsible for the resistance drift in the amorphous phase^7^.

However, pure Sb_2_Te_3_ suffers from an inadequate data retention due to insufficient stability of the amorphous phase. It would therefore be of interest to investigate the possibility to substitute Sb_2_Te_3_ with a phase change material with a higher crystallization temperature such as GST or GeTe. In this respect, we must however consider that nanoconfinement could have several possible effects on the crystallization kinetics^8^. In the case of elemental Sb, for instance, it was shown that the amorphous phase can be dramatically stabilized against recrystallization in ultrathin films 3–10 nm thick capped by insulating layers^9,10^. It is therefore important to assess whether the materials could retain a high crystallization speed at high temperature along with good data retention at low temperature when it is confined in ultrathin films.

On these premises, in this article we report on molecular dynamics (MD) simulations of the crystallization of the phase change compound GeTe in a nanoconfined geometry. The simulations aimed at addressing the effect of confinement of GeTe slabs in a superlattice made of alternating layers of GeTe and TiTe_2_ similarly to the Sb_2_Te_3_/TiTe_2_ superlattices of Ref.^7^. We have chosen GeTe because it shares many properties with the most commonly studied GST alloy and because of the availability of an interatomic potential for large scale simulations that we have previously devised in Ref.^11,12^. This potential was generated by fitting with a Neural Network (NN) method^13^ a large database of energies of small models computed within Density Functional Theory (DFT). We have not explicitly considered a real TiTe_2_ slab which would require to device a proper NN potential, but we mimicked the confinement effects of TiTe_2_ on GeTe by a suitable capping as discussed in the next section. The simulations reveal that the crystal growth velocity in the confined geometry is indeed lower with respect to the bulk, but to an extent (about a factor two at the temperature of maximum speed) which does not hinder the exploitation of GeTe/TiTe_2_ superlattice for neumorphic applications.

## Results and discussion

At normal conditions, GeTe crystallizes in a trigonal geometry (space group *R*3*m*)^14^. This phase named $\alpha$-GeTe, with two atoms per unit cell, can be viewed as a distorted rocksalt geometry with an elongation of the cube diagonal along the [111] direction and an off-center displacement of the inner Te atom along the [111] direction giving rise to a 3+3 coordination of Ge with three short and stronger bonds (2.84 Å) and three long and weaker (3.17 Å) bonds. In the conventional hexagonal unit cell of the trigonal phase, the structure can be also seen as an arrangement of GeTe bilayers along the *c* direction with shorter intrabilayer bonds and longer interbilayers bonds. The interplanar distance within a bilayer is 1.506 Å while the interplanar distance across two bilayers is 2.062 Å. The trigonal ferroelectric phase transforms into the cubic paraelectric phase ($\beta$-GeTe, space group Fm${\bar{3}}$m) above the Curie temperature of 705 K^15^. In the cubic phase, the alternation of long and short bonds survives in a disordered manner along all equivalent <111> directions as revealed by extended x-ray absorption fine structure (EXAFS), x-ray total diffraction measurements^16,17^ and MD simulations^18^. However, more recent molecular dynamics simulations^19^ suggest that the order-disorder character of the phase transition is weaker than as inferred from EXAFS data.

**Figure 1:**
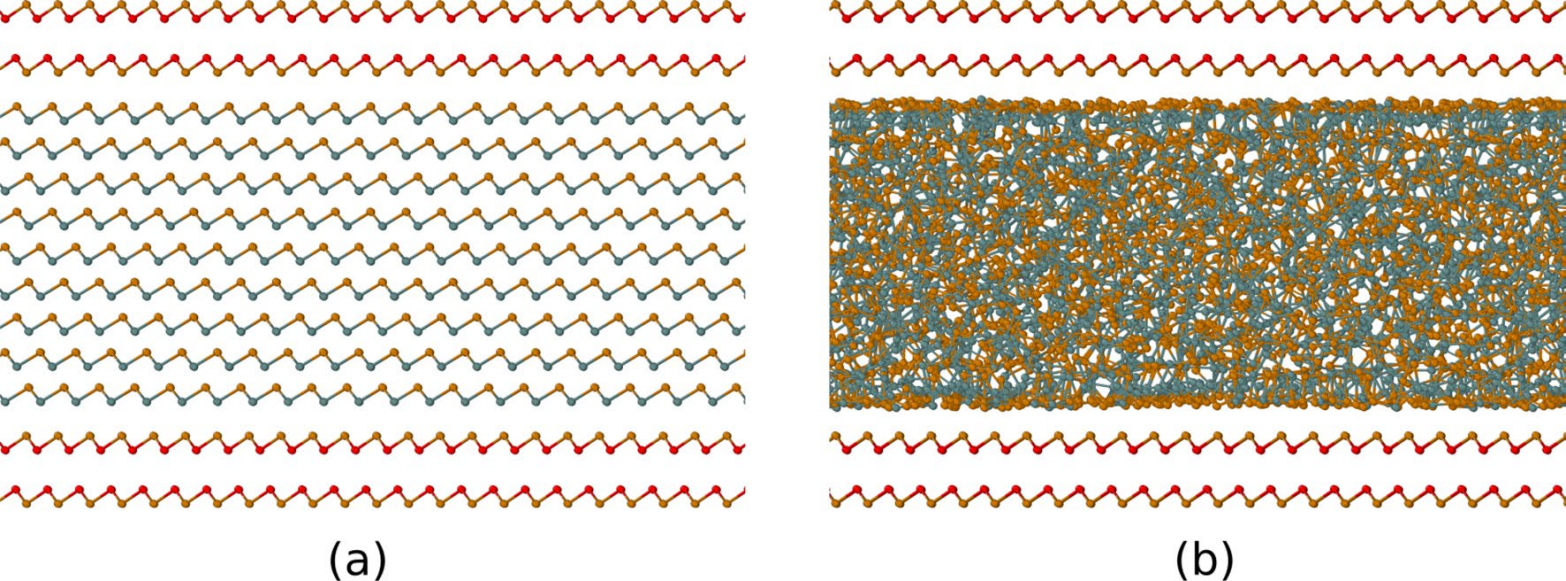
(**a**) Crystalline and (**b**) amorphous phase of the slab made of nine bilayers of $\alpha$-GeTe encapsulated by capping layers. The capping layer is made by a frozen bilayer of crystalline GeTe at the lattice constant of TiTe_2_, aiming at mimicking the confining slabs of TiTe_2_ in GeTe/TiTe_2_ superlattices. Color code for atomic spheres: Ge (gray), Te (orange), Ge atoms in the capping layers (red).

We have simulated the effect of confinement on the crystallization of GeTe by considering a slab made of nine bilayers of $\alpha$-GeTe (18 atomic planes with a thickness of about 3 nm), encapsulated by capping layers aiming at mimicking the confining slabs of TiTe_2_ in GeTe/TiTe_2_ superlattices. The capping layer mimicking TiTe_2_ on each side is made by a frozen bilayer of crystalline GeTe itself constrained at the lattice parameter of TiTe_2_ as shown in Fig. 1a. This choice is made because for the MD simulations we used a NN potential suitable to describe only Ge–Ge, Ge–Te and Te–Te interactions. In fact, TiTe_2_ is a layered hexagonal crystal (space group P${\bar{3}}$m1) made of trilayer Te–Ti–Te blocks stacked along the *c* axis and separated by van der Waals gaps. The geometry of the hexagonal Te layers is the same in TiTe_2_ and GeTe albeit with different lattice parameters, namely a = 3.7795 Å for TiTe_2_^20^ and a = 4.1677 Å for GeTe^14^. A good commensuration between a trilayer of TiTe_2_ and $\alpha$-GeTe in the hexagonal *xy* plane is obtained by considering multiples of the orthorhombic supercells with edge *a* and $\sqrt{3}a$, namely 11 x 10 orthorhombic cells of TiTe_2_ and 10 x 9 orthorhombic cells of $\alpha$-GeTe. The misfit is only 0.2 $\%$ along *x* and 0.7 $\%$ along *y*. We finally set the in-plane lattice parameters of the supercell to those of $\alpha$-GeTe which means that the bilayers mimicking TiTe_2_ are slightly strained by the amount given above. The model thus contains 10 x 9 x 2 = 180 atoms per atomic layer of GeTe and 11 x 10 x 2 = 220 atoms in each of the bilayers mimicking TiTe_2_, for a total amount of 4120 atoms. Periodic boundary conditions are applied along the three cartesian axis (see Fig. 1a). The TiTe_2_-like bilayers are oriented in such a way to expose the Te layer to the $\alpha$-GeTe slabs on both sides as it would occur for a TiTe_2_ slab. The distance between the two TiTe_2_-like bilayers is fixed to an arbitrary value of 4 Å as the two bilayers are just meant to mimic TiTe_2_ slabs encapsulating GeTe from both sides and they must not interact with each other. The distance between the TiTe_2_-like bilayers and GeTe is instead optimized by minimizing the energy with respect to the *c* axis of the supercell. However, since this is just the initial model to be subjected to a thermal cycle of amorphization/crystallization, we decided to constraint the distance between GeTe and the Te layer of the capping layers to be the same on both sides, although on one side we have a Ge–Te contact and on the other side a Te–Te contact (see Fig. 1a). This choice is made because after a thermal cycle of amorphization/crystallization, we expect that the amorphous GeTe would face in the same manner the capping layer on both sides. We will discuss further this issue later on. This procedure finally yields an interplanar distance between Te atoms of the capping layers and the outermost atomic layer of GeTe of 3.463 Å which is close to interplanar distance of 3.48 Å between TiTe_2_ and Sb_2_Te_3_ in the TiTe_2_/Sb_2_Te_3_ superlattice of Ref.^7^. We will refer to this model of GeTe encapsulated by TiTe_2_-like capping layer as the superlattice (SL) configuration. Because of the large lattice mismatch between GeTe and TiTe_2_, we assume that crystal nucleation would not be triggered by the interaction with the TiTe_2_ slabs. Under this assumption our fake TiTe_2_ would be sufficient to mimic the confinement effects introduced by TiTe_2_. As we will see in the following, crystal nucleation still occurs at the surface of the amorphous slab, not because of interaction with TiTe_2_ but because of an atomic layering at the surface of the amorphous phase.

Since TiTe_2_ has a much higher melting temperature than GeTe, we mimicked the confinement by TiTe_2_ by freezing the atoms of the crystalline GeTe-like capping bilayers during the thermal cycle. Amorphization of the GeTe slab was achieved by equilibrating the system first at 1500 K for 200 ps and then at 1000 K for 100 ps. The liquid-like slab was then quenched to 300 K in 100 ps.

A snapshot of the amorphous GeTe (a-GeTe) slab encapsulated by the frozen capping bilayers mimicking TiTe_2_ (superlattice configuration) is shown in Fig. 1b. We observed a small expansion of the amorphous slab which leads to a density of about 0.0363 atom/Å^3^ to be compared with the initial density of 0.0372 atom/Å^3^ of the slab in the crystalline phase. Because of the constraints imposed by the capping layers, the density of the amorphous slab is, however, sizeably larger than the theoretical equilibrium density of a-GeTe of 0.0315 atom/Å^3^ given by the NN potential^21^. We mention that the theoretical density of a-GeTe computed with the NN potential (and consistently with the DFT framework)^21^ is lower than the experimental value of 0.03327 atom/Å^3^^22^.

**Figure 2:**
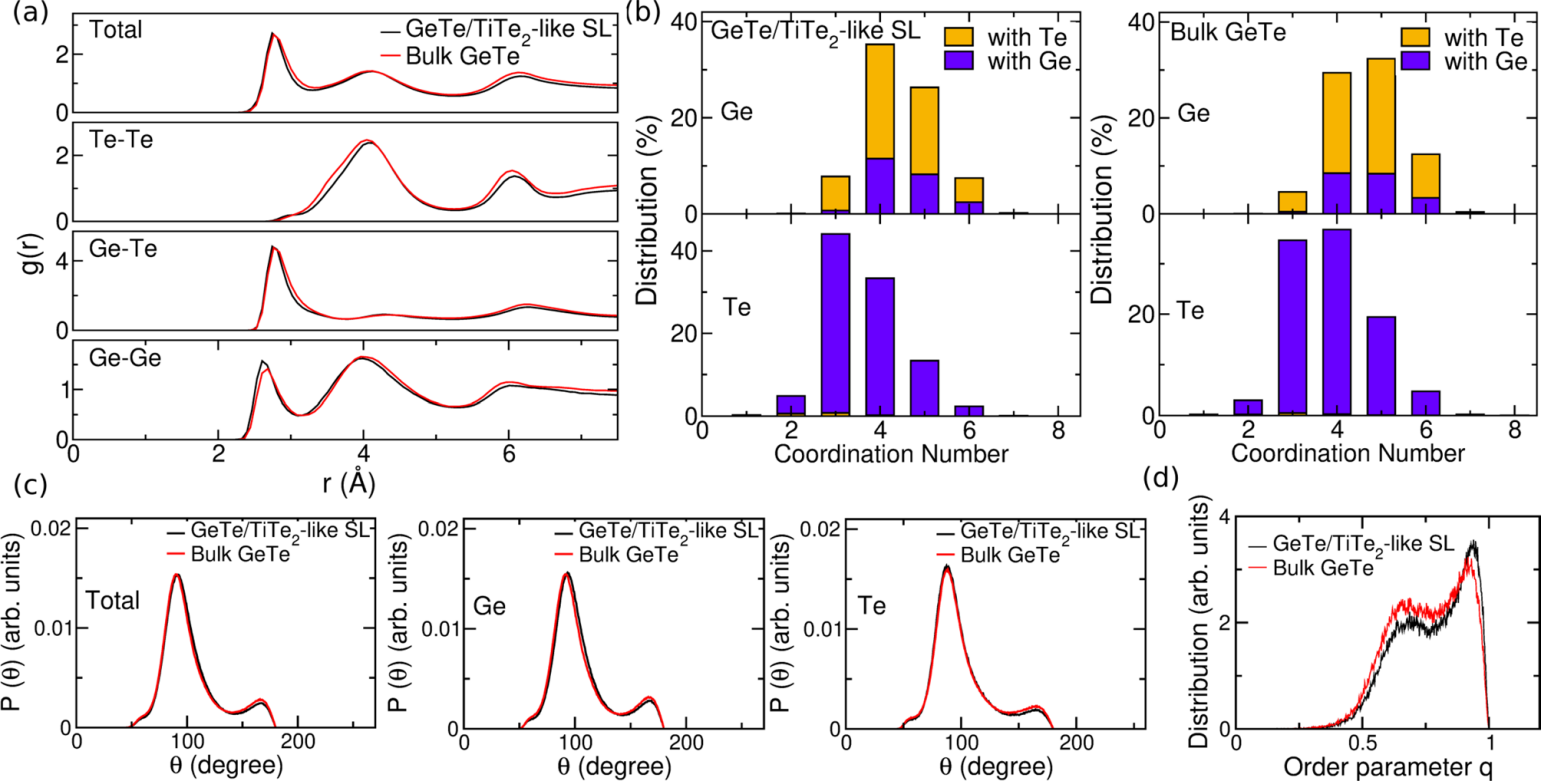
Structural properties at 300 K of the slab of amorphous GeTe (black lines, see Fig.1b) confined by the TiTe_2_-like capping layers compared to those of a bulk model of amorphous GeTe at the density of $\alpha$-GeTe generated by quenching from the melt (red lines). (**a**) Partial pair correlation functions. (**b**) Distribution of coordination numbers resolved per chemical species obtained by integrating the partial pair correlation functions up to the bonding cutoff of 3.00 Å for Ge–Ge and Te–Te bonds and 3.22 Å for Ge–Te bonds. (**c**) Bond angle distribution function resolved per central atomic species. The data are normalized to the number of triplets in each model. (**d**) Distribution of the *q* order parameter for tetrahedricity of the fourfold coordinated Ge atoms.

We compared the structural properties of the amorphous phase to those of a bulk model quenched from the melt at the density fixed to that of the crystalline $\alpha$-GeTe of 0.0372 atoms/Å^3^. Structural properties of the resulting amorphous slab were computed over 40 ps simulation at 300 K. Partial pair correlation functions, bond angle distribution functions, and distribution of the coordination numbers for the a-GeTe slab are compared with the results for the bulk in Fig. 2. The average partial coordination numbers of the slab and the bulk are compared in Table 1. The coordination numbers are obtained by integrating the partial pair correlation functions up to the bonding cutoff of 3.00 Å for Ge–Ge and Te–Te bonds and 3.22 Å for Ge–Te bonds. The coordination numbers are lower in the superlattice than in the bulk in part because of the slightly lower density (0.0363 atom/Å^3^ vs. 0.0372 atom/Å^3^). However, there is a further reduction in the coordination number of Te because of an enrichment of Te at the two surfaces of the amorphous slab facing the Te planes of the capping layers. This feature is reproduced also by repeating the simulation for two different models with a shorter distance between the Te plane of the capping layer and the outermost plane of the GeTe block (see Fig. S1 in the Supplementary Information). In a first model, we changed the orientation of the TiTe_2_-like GeTe bilayers in such a way that the GeTe slab faces a Te capping layer on one side and a Ge capping layer on the other side both at a distance of 3.03 Å. In a second model, the interplanar distances between the Te layer of the TiTe_2_-like GeTe bilayer and the Ge and Te layers on the two side of the GeTe slab were obtained from DFT optimization of a real GeTe/TiTe_2_ SL^23^ resulting in a value of 3.4 Å for Te–Te interplanar distance and of 2.74 Å for Ge–Te interplanar distance.

**Table 1:** Average coordination number for different pairs of atoms computed from the partial pair correlation functions for the amorphous slab confined by the capping layers, compared with the data of a bulk amorphous model at the density of crystalline $\alpha$-GeTe (see text).

	Bulk GeTe	GeTe/TiTe_2_
Ge		
with Ge	0.994	0.991
with Te	3.599	3.331
Total	4.543	4.322
Te		
with Ge	3.599	3.331
with Te	0.038	0.057
Total	3.637	3.388

As discussed in several previous works^21,24,25^, Te atoms are mostly three-fold coordinated in a pyramidal geometry, most of the Ge atoms are three-fold coordinated in a pyramidal geometry and four- or five-fold coordinated in a defective octahedral environment (octahedral bonding angles but coordination lower than six), while a minority fraction of Ge atoms are in tetrahedral geometries. The tetrahedral configuration is favored by homopolar Ge–Ge bonds^26^. These structural features are revealed by the bond angle distribution function (Fig. 2c): the peak at about 90^o^ in the angle distribution function of Ge atoms is due to pyramidal and defective octahedral configurations, the weak peak at about 170^o^ is due to axial bonds in a defective octahedral configuration and the shoulder at about 109^o^ is due to tetrahedra. A quantitative measure of the fraction of tetrahedral environments can be obtained from the local order parameter *q* introduced in Ref.^27^. It is defined as $q = 1-\frac{3}{8}\sum _{i > k} (\frac{1}{3} + \cos \theta _{ijk})^2$, where the sum runs over the pairs of atoms bonded to a central atom *j* and forming a bonding angle $\theta _{ijk}$. The order parameter evaluates to *q* = 1 for the ideal tetrahedral geometry, to *q* = 0 for the 6-fold coordinated octahedral site, to *q* = 5/8 for a 4-fold coordinated defective octahedral site, and *q* = 7/8 for a pyramidal geometry (three bonding angles of 90^∘^). The distribution of the *q* order parameters for the slab and bulk models are compared in Fig. 2d. As discussed in previous works^28–30^, the fraction of Ge atoms in tetrahedral environments can be quantified by counting the 4-fold coordinated atoms with *q* in the range $0.8<q<1$. The resulting fraction of Ge atoms in tetrahedral geometry (with respect to the total number of Ge atoms) is 25 $\%$ in the slab and 19 $\%$ in the bulk. The coordination numbers of bulk a-GeTe at the experimental density of the crystalline $\alpha$ phase are larger than those of a-GeTe at the experimental density (0.03327 atoms/Å^3^) as expected due to the density increase (see for instance Ref.^21^).

**Figure 3:**
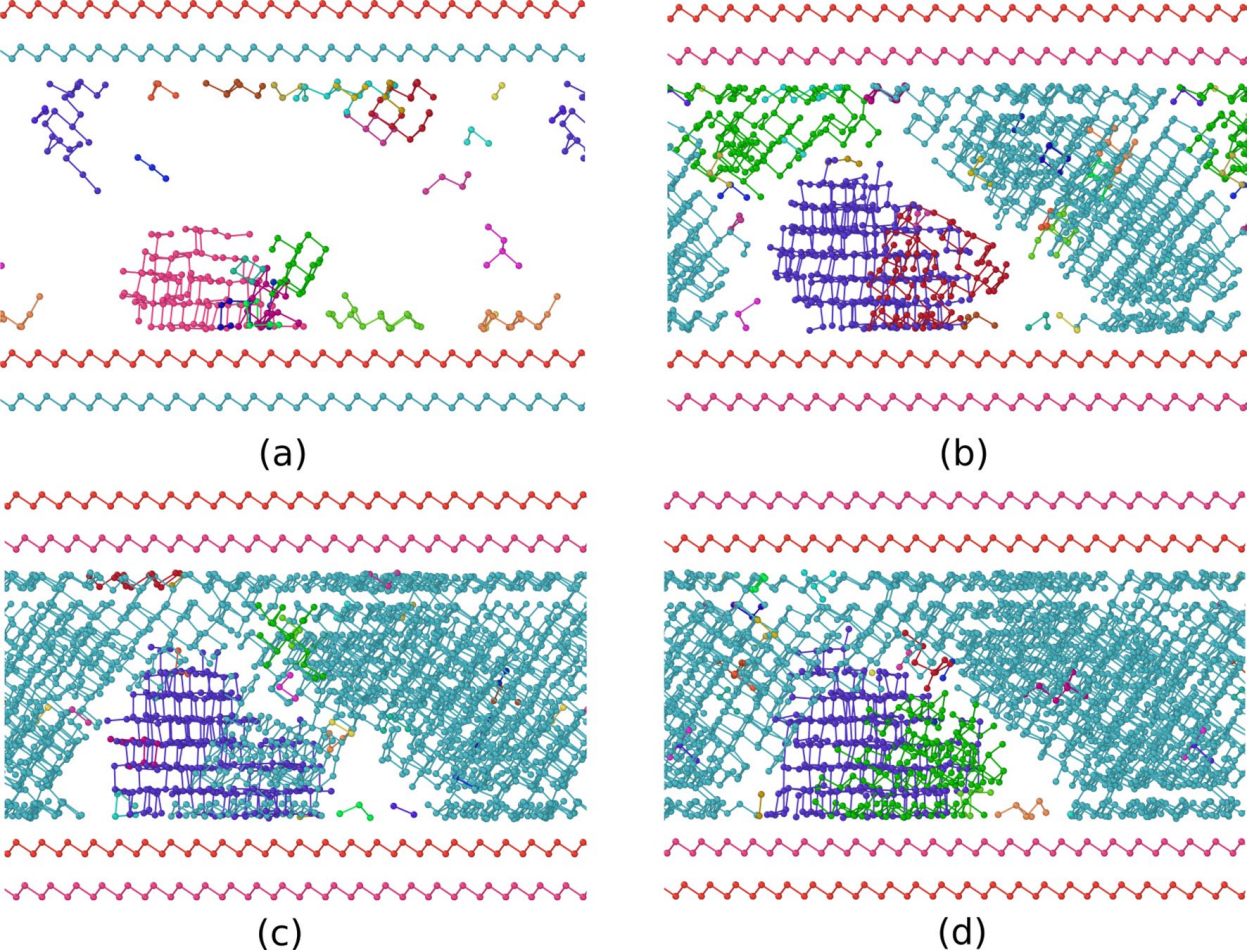
Simulation of the crystallization of a 3240-atom slab of amorphous GeTe capped by bilayers mimicking confinement by TiTe_2_ in GeTe/TiTe_2_ superlattices. Snapshots at different times at 600 K are shown for (**a**) 0.25 ns (**b**) 0.5 ns (**c**) 0.75 ns and (**d**) 1 ns. Crystallization starts at the surfaces of the amorphous slab, albeit the capping layers do not act as nucleation centers. Only crystalline atoms, identified by the $Q_4^{dot}$ order parameter (see Methods), are shown. Different crystalline nuclei have different colors.

**Figure 4:**
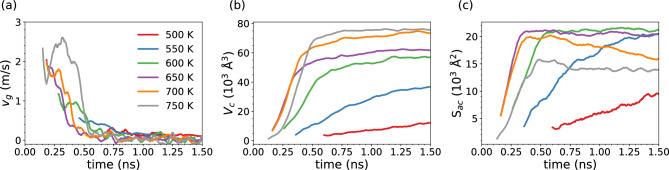
(**a**) Instantaneous crystal growth velocity $v_g$, (**b**) volume occupied by the crystalline atoms $V_c$ and (c) area of the crystal-amorphous interface $S_{ac}$ as a function of time at the different temperatures in the crystallization of the superlattice configuration (GeTe/TiTe_2_-like SL). The crystal growth velocity is computed as $v_g = dV_c/dt S_{ac}^{-1}$ as described in Refs.^31^. The $v_g$ reported in Table 2 are obtained by averaging the instantaneous $v_g$ over the time intervals highlighted in Fig. S5 in the Supplementary Information.

The amorphous model was then heated at six different target temperatures of 500, 550, 600, 650, 700, and 750 K to study the crystallization process, which was monitored in constant volume simulations, 1 ns long, at each temperature. The number of crystalline atoms was identified by the local order parameter $Q_{4}^{dot}(i)$ (see Methods). Crystal nucleation starts at both surfaces of the amorphous slab, albeit the capping layers do not act as nucleation centers since there is no registry with the lattice of GeTe.

Snapshots of the crystallization process at 600 K are shown in Fig. 3; similar snapshots for the simulations at 650 and 700 K are given in Fig. S2 and Fig. S3 in the Supplementary Information. In all simulations we observed the formation of several crystalline nuclei. At the lower temperatures of 550–650 K, the final configuration is a polycrystalline model with several grains. At 700 and 750 K we observed the coarsening of different grains leading to a final single crystal model. The coarsening of the nuclei with different crystallographic orientation is a much slower process than the nucleation and growth of individual nuclei. Therefore, we observed coarsening only at the higher temperatures (700 and 750 K) because there the number of different nuclei is smaller due to the lower nucleation rate (the size of the critical nucleus is larger) and because the coarsening process is accelerated by temperature. A movie of the crystallization and coarsening processes at 700 K is provided as Supplementary Information.

As already mentioned, the nucleation started at the surfaces, possibly because of a sort of layering due to the excess of Te on the outermost planes. Because of that, in the final configuration at 700 and 750 K where a single grain is formed, a Te layer is present at both surfaces which leads to the formation of 19 atomic layers instead of the 18 layers of the starting crystalline GeTe slab. As a consequence, several antisite defects (because of the Te plane is excess) and vacancies are present in the crystallized model. At all temperatures in the range 550–750 K, the crystalline phase is cubic-like as the $\beta$-phase of GeTe. By quenching to 300 K, the crystalline grains still keep a disordered $\beta$-phase configuration possibly because of a large number of defects. This occurs for all the models crystallized at different temperatures.

**Table 2:** Crystal growth velocities $v_g$ (m/s) computed as described in Methods for the GeTe slab confined by the TiTe_2_-like capping layers (GeTe/TiTe_2_-like SL) for the bulk models (see text) with a thick (28 layers, Bulk28) molten region in contact with a crystalline slab and from homogeneous crystal nucleation and growth (Bulk_homo_). At 700 and 750 K no crystal nucleation occurs in the bulk over the time scale of several ns.

Temperature (K)	GeTe/TiTe_2_-like SL	Bulk28	Bulk_homo_
	$$v_g$$ (m/s)		
500	0.1	0.5	0.7
550	0.4	1.3	1.4
600	0.9	3.9	2.4
650	1.6	4.0	4.5
700	1.7	3.7	–
750	2.3	3.4	–

The number of crystalline atoms as a function of time for the simulations at different temperatures is shown in Fig. S4 in the Supplementary Information. The crystal growth velocity $v_g$ has been computed from the time derivative of the crystalline volume $V_c$ according to the scheme proposed in Ref.^32^ as $v_g(t)$ = $S_{ac}^{-1}$d$V_c$/dt where $S_{ac}$ is the area of the crystal-amorphous interface (see Methods). The crystal growth velocity was calculated at different temperatures, after equilibration at the target temperature and before the different nuclei start to coalesce. The instantaneous $v_g$, $V_c$ and $S_{ac}$ (see Ref.^31^) as a function of time at the different temperatures is shown in Fig.  4. The average crystal growth velocities are obtained by averaging the instantaneous crystal growth velocity in a time interval of a few hundreds of ps as shown in Fig. S5 in the Supplementary Information. The resulting $v_g$ are compared in Table 2 with those of reference calculations for the bulk. We considered different models for the bulk amorphous phase at the density of crystalline $\alpha$-GeTe. We first considered a slab of amorphous GeTe in contact with a slab of crystalline GeTe. The model was prepared starting from an orthorhombic cell of crystalline $\alpha$-GeTe at the crystalline equilibrium density (0.0372 atoms/Å^3^) consisting of 54 atomic layers with 180 atoms per layer. The system was then brought to 1500 K for 200 ps by letting 28 layers free to move and the other 26 frozen (model Bulk28 in Table 2). The molten region was then equilibrated at 1000 K for 100 ps and then quenched at 300 K in 100 ps, then all the atoms have set free to move and the system was equilibrated at the different target temperatures to compute the crystal growth velocities along the growth direction corresponding to the *c* axis of $\alpha$-GeTe.

Moreover, we considered also a cubic 4096-atom bulk model of the amorphous phase still at the density of crystalline $\alpha$-GeTe to study homogeneous crystal nucleation and growth. The number of crystalline atoms as a function of time at different temperatures for the two bulk models of a-GeTe are shown in Fig. S4 in in the Supplementary information. In the range 500-600 K, we see the formation of several overcritical nuclei, while at 650 K a single single nucleus forms and no nucleation occurs at and above 700 K up to 2 ns. The instantaneous $v_g$, $V_c$ and $S_{ac}$ (see Ref.^31^) as a function of time at the different temperatures is shown for the two bulk models in Fig. S6 and S7 in the Supplementary information.

The average crystal growth velocities $v_g$ for the two bulk amorphous models are reported in Table 2. The results on $v_g$ for the bulk-like models are similar and about a factor two larger than those of the slab confined by the frozen TiTe_2_-like capping layers at the temperatures at maximal crystallization speed. The difference between the $v_g$ in the slab and in the bulk decreases by increasing temperature. The lower $v_g$ in the slab can be ascribed to the interaction among several nuclei which is enhanced with respect to the bulk because in the slab the nucleation centers appear just at the two surfaces. This means that the different nucleation centers are on average closer in the slab than in the bulk. There is a small reduction of the atomic mobility in the slab with respect to the bulk (see Table S1 and Fig. S8 in the Supplementary Information) which is, however, not sufficiently large to account for the difference in $v_g$ between the bulk and the slab.

Note that the crystal growth velocities of Table 2 for the bulk models are lower than those reported in previous simulations with the same NN potential because here the density is set to that of the $\alpha$-GeTe which is higher than the experimental density of the amorphous phase considered in previous works^12^. A dependence of the crystal growth velocity on the density was also highlighted in previous works^33,34^ (see for instance Fig. 2a in Ref.^33^).

In the crystal growth from the amorphous/crystal interface in the bulk (Fig. S6 in the Supplementary Information), one observes an oscillation in time of the instantaneous crystal growth velocities. This can be ascribed to a different sticking coefficient of the Te and Ge atoms at the crystalline surface. In fact, the growth along the *c* axis of $\alpha$-GeTe corresponds to the formation of the alternating layers made of only one atomic specie. The oscillation periodic is shorter than the time needed to complete a single crystalline layer because it results from the superposition of two growing surfaces. The same effect is also present at intermediate temperatures in the slab model (see Fig. S5 in the Supplementary Information).

Overall, we can conclude that the confinement has not a dramatic effect on the crystallization kinetic of our models of GeTe/TiTe_2_-like superlattice. Although a reduction in the crystal growth velocity with respect to the bulk is indeed observed at the lower temperatures, the maximum crystallization speed of the confined GeTe slab is comparable to that of the bulk at the same density which makes the GeTe/TiTe_2_ superlattice a viable candidate for applications in neuromorphic computing with a foreseen improvement in data retention with respect to the Sb_2_Te_3_/TiTe_2_ SL proposed in Ref.^7^.

**Figure 5:**
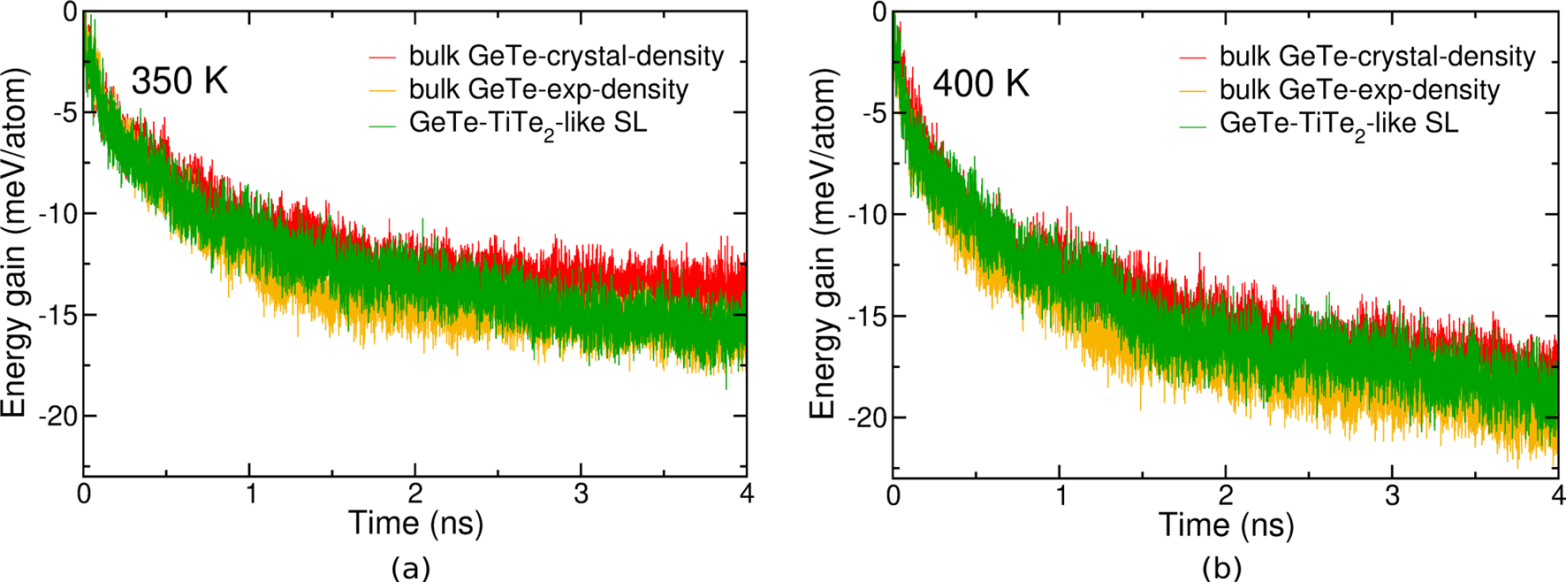
Energy gain per atom as a function of time due to structural relaxations in NVT simulations at (**a**) 350 K and (**b**) 400 K for a-GeTe in a bulk model at the crystal density (red line), a-GeTe in a bulk model at the experimental density (orange line) and in confined slab geometry of the GeTe/TiTe_2_-like superlattice (green line).

Another advantage of the confinement in a SL geometry reported for Sb_2_Te_3_/TiTe_2_ in Ref.^7^ is the reduced drift of the electrical resistance in the reset state due to reduced structural relaxations, i.e. aging, of the amorphous phase in confined ultrathin (about 5 nm thick) films. The same effect was also reported in Ref.^9^ for ultrathin films of Sb (3–10 nm thick) confined by capping layers. It is therefore interesting to address whether a similar behavior could be observed in the GeTe/TiTe_2_ superlattices. The drift in the electrical resistance is typically monitored on time scales (from seconds to hours)^7,35^ not accessible by MD simulations. Nevertheless, we can attempt to estimate the effect of confinement on the structural relaxations in our models of the amorphous phase which are very far from the ideal glass because they are generated by fast quenching from the melt (100 ps). To this aim, starting from the models of bulk a-GeTe (4096-atom cubic box) and of the confined amorphous slab (about 3 nm thick) in the GeTe/TiTe_2_-like superlattice equilibrated at 300 K, we raised the temperature to 350 or 400 K in NVT simulations to accelerate the structural relaxations by still keeping the system below the glass transition to prevent crystal nucleation and growth. We considered models of bulk a-GeTe both at the experimental density of a-GeTe and the density of crystalline $\alpha$-GeTe (see above). The energy gain due to the structural relaxations is then monitored as a function of time up to 4 ns as shown in Fig. 5. Actually, we do not observe sizeable differences in the behavior of a-GeTe in the bulk and in the confined slab which would suggest that the drift in a-GeTe is not mitigated by confinement in the geometry chosen here. The slightly faster structural relaxation in the sequence of bulk at the experimental density, GeTe/TiTe_2_ SL and bulk at the crystal density is consistent with an acceleration of the drift by decreasing the density of the amorphous phase. We must, however, emphasizes once more that the time scale considered here for the structural relaxations (4 ns) is much shorter than the time scale over which the drift in the electrical resistance is measured experimentally for instance in the Sb_2_Te_3_/TiTe_2_ superlattice of Ref.^7^ (from seconds to hours), albeit the experimental data refer to 300 K while our simulations are performed at 350–400 K.

## Conclusions

In summary, we performed MD simulations on the crystallization kinetics of ultrathin (3 nm) slabs of GeTe confined by capping layers aimed at mimicking the TiTe_2_ spacers in so far hypothetical GeTe/TiTe_2_ superlattices. This system is analog to the Sb_2_Te_3_/ TiTe_2_ heterostructure proposed in Ref.^7^ for applications in neuromorphic devices. The replacement of Sb_2_Te_3_ by GeTe would raise the crystallization temperature of the amorphous phase to improve the data retention. Since nanoconfinement is known to slow down the crystallization kinetics, for instance in elemental Sb^9^, we have investigated here nanoconfined GeTe to explore its potential applications in GeTe/TiTe_2_ superlattices. The simulations show that nanoconfinement leads to a decrease of the crystal growth velocity $v_g$ with respect to the bulk amorphous phase at the same density, set here to that of the crystalline $\alpha$ phase. The reduction in $v_g$, about a factor two at the temperature of maximal crystallization speed, is however rather minor in the perspective application in neuromorphic devices. As opposed to what found experimentally for Sb_2_Te_3_ and elemental Sb^7,9^, we do not observe a reduction of structural relaxations, i.e. aging, in nanoconfined GeTe with respect to the bulk. However, we must remark that we have investigated structural relaxation in the amorphous phase on a time scale (4 ns at 400-350 K) much shorter than that considered experimentally to monitor the resistance drift (from seconds to hours at 300 K) in Sb_2_Te_3_/TiTe_2_ superlattices^7^ and in ultrathin GeTe layers (3 nm) in memory cells^35^. Therefore, we can not exclude that a mitigation of the drift could also be achieved in nanoconfined GeTe on the time scale of interest for the operation of the devices. In conclusions, MD simulation support the idea of investigating GeTe/TiTe_2_ superlattices for applications in neuromorphic devices with improved data retention that we here put forward to future experimental work.

## Methods

Molecular dynamics (MD) simulations have been performed by using the NN interatomic potential for GeTe developed in Ref.^11,12^. The potential was originally obtained in Ref.^11^ by fitting a database of total energies obtained within DFT by means of the method introduced by Behler and Parrinello^13^. The database consists of the DFT energies of about 30000 configurations of 64-, 96-, and 216-atom supercells computed by employing the Perdew–Burke–Ernzerhof (PBE) exchange and correlation functional^36^ and norm conserving pseudopotentials. In order to deal with surfaces and nanowires, a new version of the potential was generated in Ref.^12^ by enlarging the training set with about 5000 new configurations of crystalline and amorphous GeTe (a-GeTe) in a slab geometry (128-atom supercell) and with about 7000 additional configurations of crystalline, amorphous and liquid GeTe in a nanowire geometry (120- and 256-atom cells). The accuracy of the NN potential in reproducing energy and forces of the training and test datasets has been discussed in Refs.^11,12^. The transferability of the potential was validated in previous works on the simulation of crystallization and thermal conductivity in the bulk and in nanowires and of the aging of the amorphous phase^33,37–41^. The potential was also recently used to simulate the deposition of GeTe films mimicking the conditions of magnetron sputtering growth^21^. MD simulations were performed with the NN code RuNNer^42^ by using the DL_POLY code as molecular dynamics driver^43^. The time step was set to 2 fs, and temperature was controlled with a stochastic thermostat^44^. To identify the crystalline nuclei we used the local order parameter $Q_{4}^{dot}(i)$^45,46^, with a slightly different definition with respect that used in our previous work^12^, given for each atom *i* by

$$\begin{aligned} Q_{4}^{dot}(i) = \frac{1}{N_i}\sqrt{\sum _{j=1}^{N_i} \sum _{m=-4}^{4}q_{4m,i} q^*_{4m,j}} \quad \quad \text {with} \quad \quad q_{4m}(i) = \frac{1}{N_i} \sum _{j=1}^{N_i} Y_{4m}(\hat{{{\textbf {r}}}}_{ij}), \end{aligned}$$

where $Y_{4m}(\hat{{{\textbf {r}}}}_{ij})$ are the spherical harmonics of the polar angles defined by the versor $\hat{{{\textbf {r}}}}_{ij}$ which links atoms *i* and *j*. The index *j* runs over the $N_i$ neighboring atoms within the cutoff of 3.2 Å. We define as crystalline an atom with $Q_{4}^{dot}$ $> 0.8$ which is a threshold suitable to discriminate a crystalline from an amorphous/liquid environment as shown in Ref.^11^ (see Fig. S9 in the Supplementary Information). Two crystalline atoms are considered connected up to a cutoff distance of 3.6 $\text{\AA }$. These choices ensure that atoms at the interface between the nuclei and the disordered phase are also considered as crystalline. The crystal growth velocity $v_g$ has been computed from the time derivative of the crystalline volume $V_c$ according to the scheme proposed in Ref.^32^ as $v_g(t)$=$S_{ac}^{-1}$d$V_c$/dt where $S_{ac}$ is the area of the crystal-amorphous interface. The crystalline volume $V_c$ is obtained by summing up the volumes of the Voronoi polyhedra of each crystalline-like atom (excluding the volume of isolated atoms or clusters of less than 28 crystalline-like atoms). $S_{ac}$ is computed as the total area of the faces that are shared by Voronoi polyhedra of amorphous-like and crystalline-like atoms. We used the Voro++ code^47^. The data of volumes $V_c$ and areas $S_{ac}$ were smoothed using a Savitzky–Golay filter with a time window of 10 = 50 ps for the calculation of growth velocity, similarly to Ref.^31^. We used the Ovito^48^ tool for the visualisation and the generation of all atomic snapshots of this manuscript.

### Supplementary Information

Supplementary Information 1.

Supplementary Information 2.

## Data Availability

The trajectory files and the OVITO files for visualization for the crystallization process at 600 and 700 K are available on the Materials Cloud repository via 10.24435/materialscloud:5k-vh. Other data that support the findings of this study are available from the corresponding author upon reasonable request.
