# Supplementary material for: Crystallization kinetics of nanoconfined GeTe slabs in GeTe/TiTe2-like superlattices for phase change memories
Source: Sci Rep. 2024 Feb 8;14:3224. doi: 10.1038/s41598-024-53192-z (PMC10853215; doi:10.1038/s41598-024-53192-z)
Supplement: Supplementary file 1 — Supplementary Information 1. [file 41598_2024_53192_MOESM1_ESM.pdf]

Crystallization kinetics of nanoconfined GeTe slabs in GeTe/$TiTe_2$-like superlattices for phase change memories
Supplementary Information

Debdipto Acharya[1], Omar Abou El Kheir[1], Davide Campi[1], and Marco Bernasconi[1,*]

[1]Department of Materials Science, University of Milano-Bicocca, Via R. Cozzi 55, I-20125 Milano, Italy
[*]marco.bernasconi@unimib.it

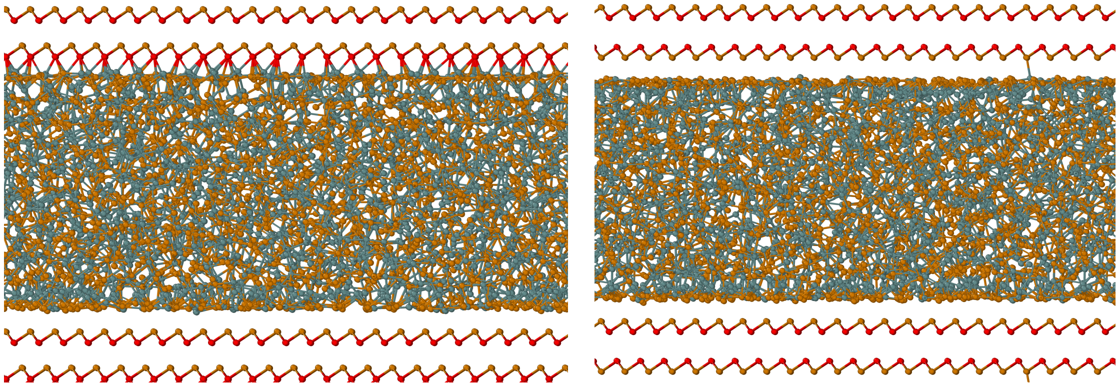

(a) (b)

Figure S1. Two models of the amorphous phase of a slab initially made of nine bilayers of α-GeTe encapsulated by capping layers. The capping layers are made of a frozen bilayer of crystalline GeTe at the lattice constant of $TiTe_2$ aiming at mimicking the confining slabs of $TiTe_2$ in GeTe/$TiTe_2$ superlattices. (a) In the first model, the $TiTe_2$-like bilayer was oriented in such a way that Ge on one side and Te on the other side face the crystalline GeTe block with a interplanar distance between the capping layer and the outermost plane of the GeTe block of 3.03 Å. (b) In the second model, the $TiTe_2$-like bilayer was oriented in such a way as to expose the Te layer to the crystalline GeTe block on both sides. The distance between the capping layer and outermost layer of GeTe slab is 3.40 Å for the Te layer and 2.74 Å for the Ge layer. The color code is the same of Fig. 1 in the article.

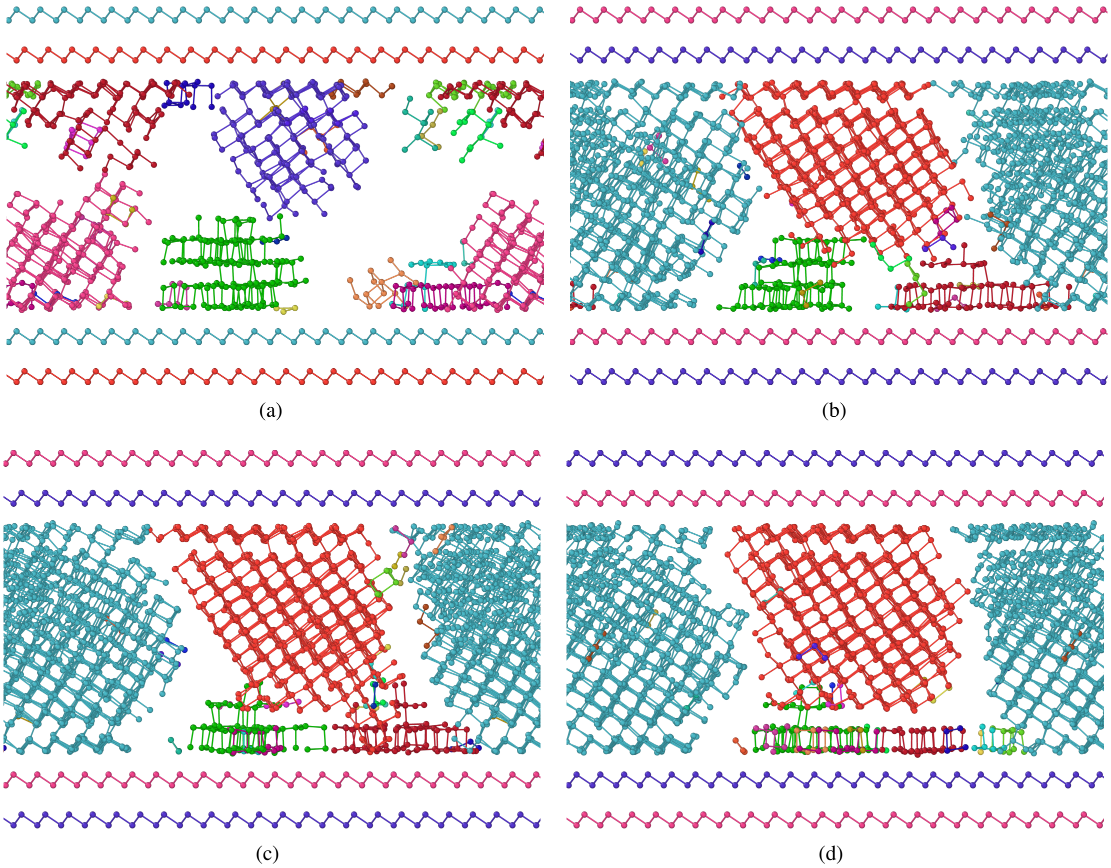

(a) (b) (c) (d)

Figure S2. Snapshots of crystalline atoms of the GeTe/$TiTe_2$-like superlattice identified by the bond parameter Q_4^{dot} at 650 K. Different crystalline nuclei are shown with different colors. The snapshots are shown for (a) 0.25 ns (b) 0.5 ns (c) 0.75 ns and (d) 1 ns.

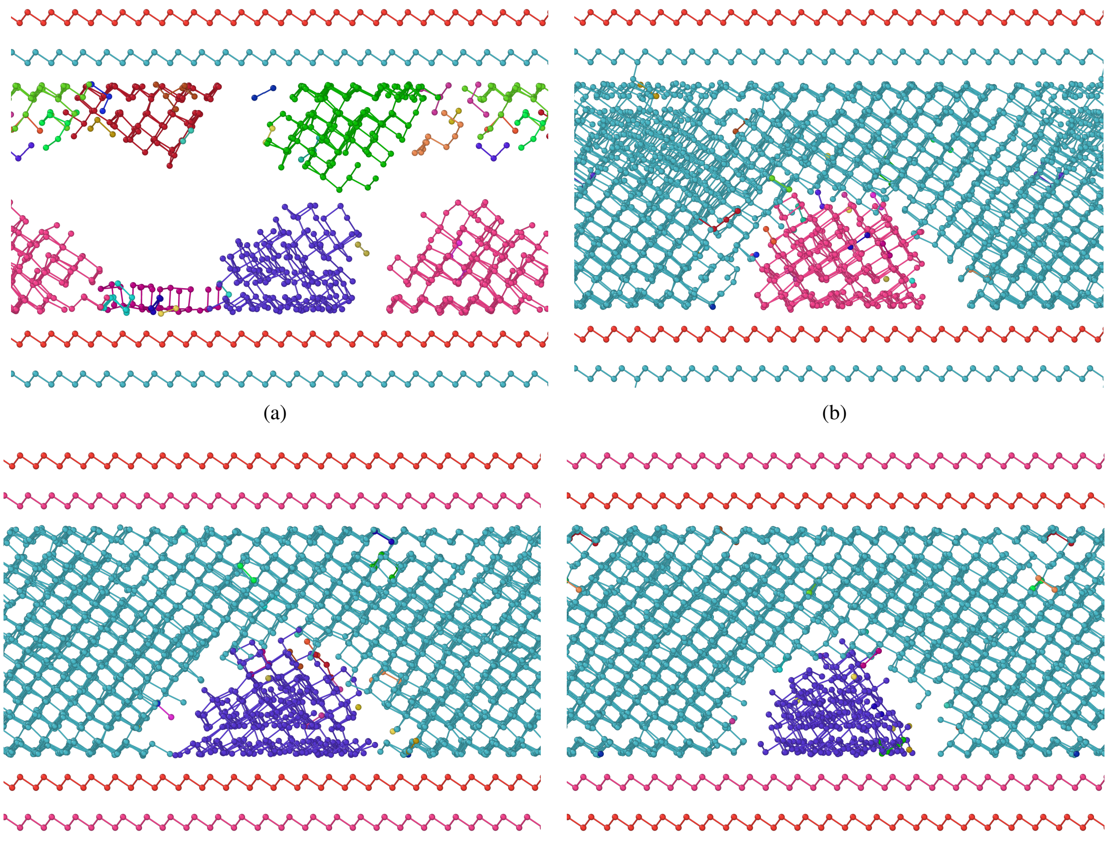

Figure S3 panel labels: (a), (b), (c), (d)

Figure S3. Snapshots of crystalline atoms of the GeTe/$TiTe_2$-like superlattice identified by the bond parameter Q_4^{dot} at 700 K. Different crystalline nuclei are shown with different colors. The snapshots are shown for (a) 0.25 ns (b) 0.5 ns (c) 0.75 ns and (d) 1 ns.

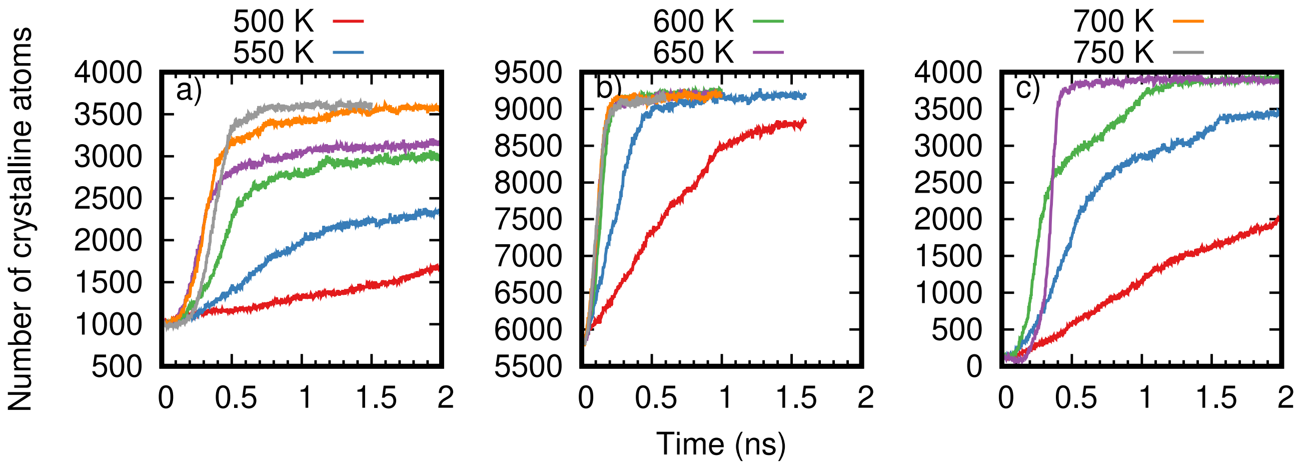

Figure S4. Number of crystalline atoms identified with the order parameter Q_4^{dot} (see article) as a function of time at different temperatures for (a) GeTe/$TiTe_2$-like superlattice (b) bulk a-GeTe (model Bulk28, see article) with crystal growth from the crystal/liquid interface and (c) bulk a-GeTe with homogeneous crystal nucleation and growth.

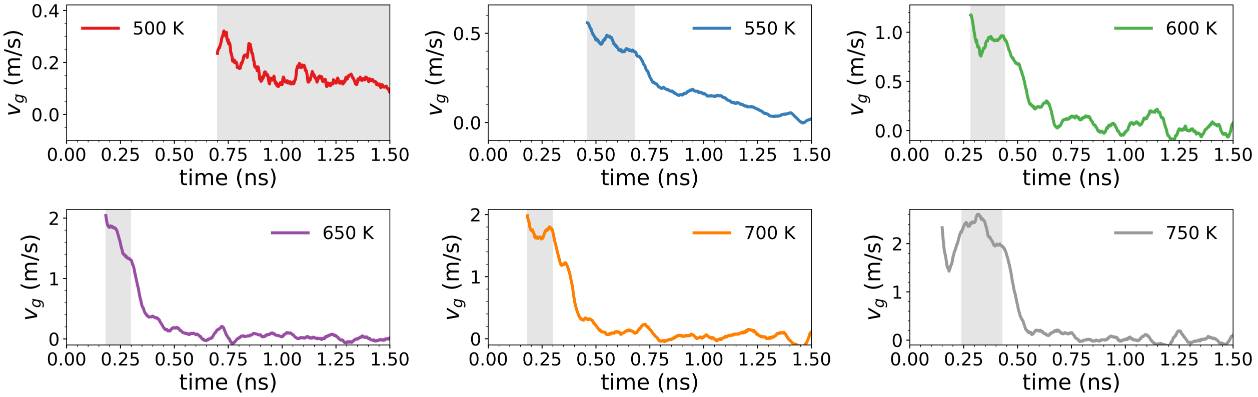

Figure S5. Instantaneous crystal growth velocity as a function of time for the model of the GeTe/$TiTe_2$-like superlattice at different temperatures. The region highlighted in gray corresponds to the time interval over which we estimated the average crystal growth velocities reported in Table 2 in the article. We start computing the instantaneous velocity when all the crystalline nuclei become overcritical.

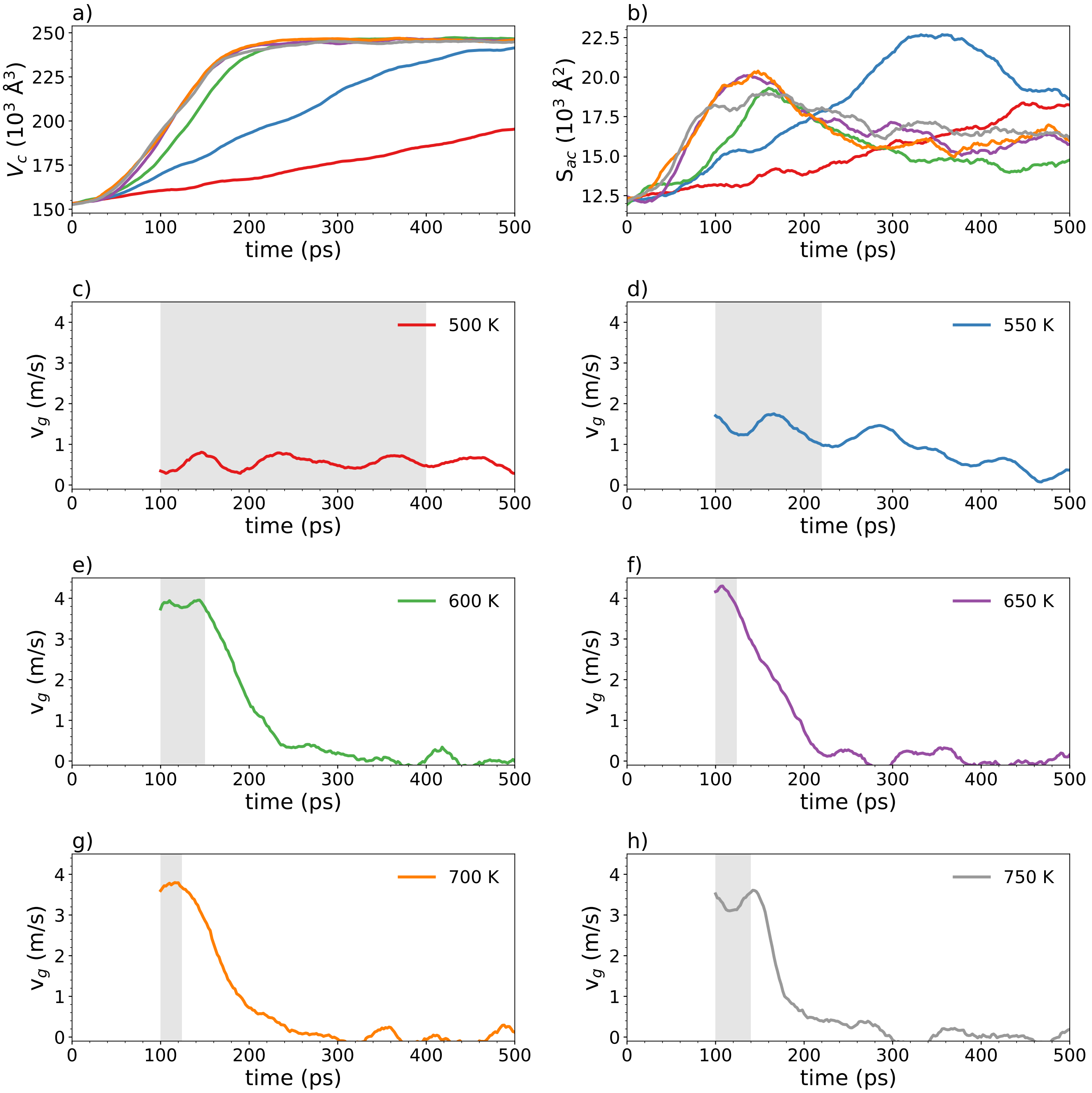

Figure S6. (a) The evolution in the time of the crystalline volume (V_c) and of (b) the area of the amorphous-crystal interface (S_{ac}) at different temperatures in bulk a-GeTe for crystal growth from the amorphous/crystal interface (model Bulk28, see article). (c)-(h) instantaneous crystal growth velocities computed as $v_g = dV_c/dtS_{ac}^{-1}$ as described in Ref. 1. The region highlighted in gray in panels (c)-(h) corresponds to the time interval over which we estimated the average crystal growth velocities reported in Table 2 in the article. We started computing v_g at a later time after proper thermalization of the system.

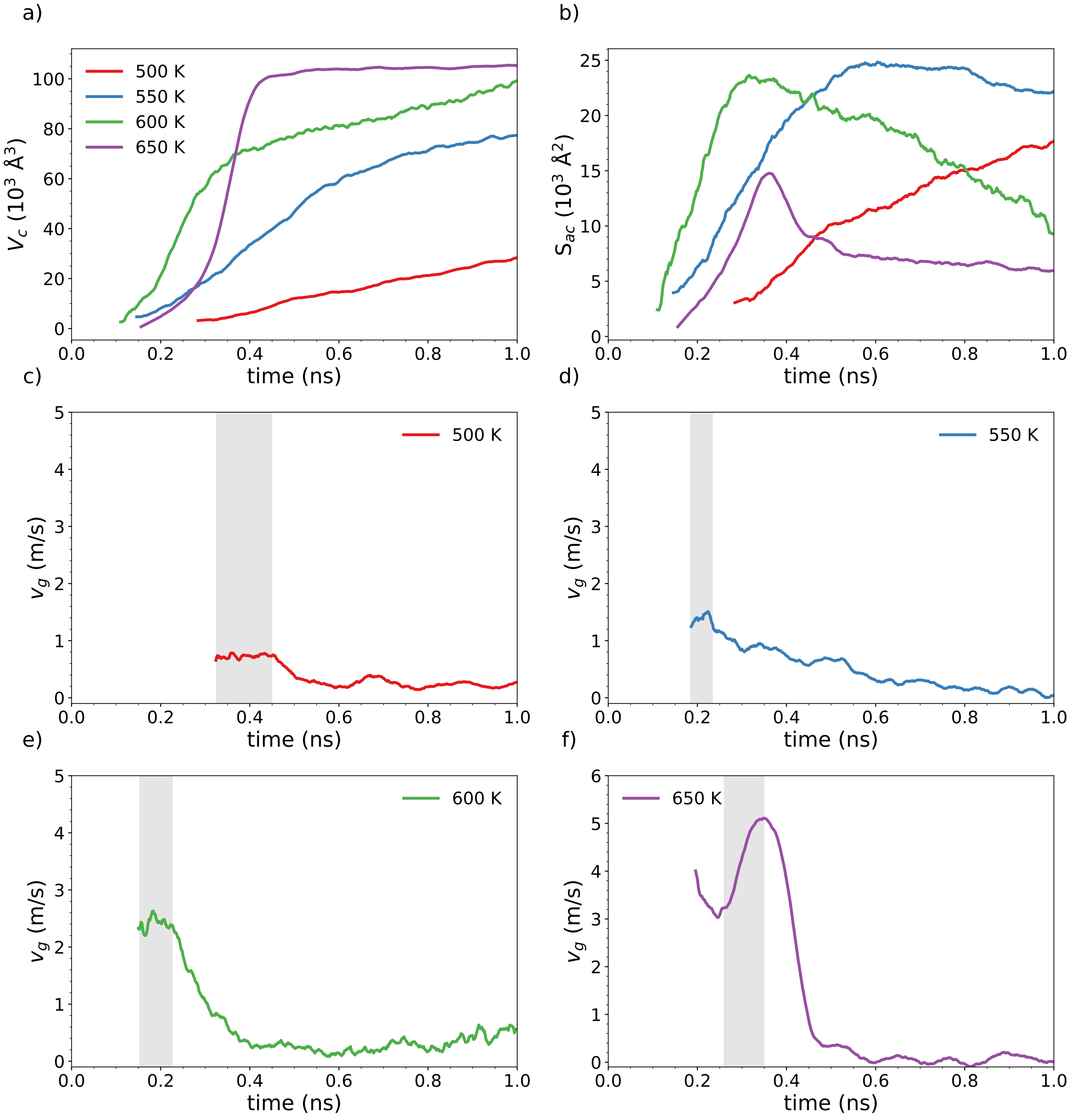

Figure S7. (a) The evolution in time of the crystalline volume (V_c) and of (b) the area of the amorphous-crystal interface (S_{ac}) at different temperatures in the 4096-atom model of bulk a-GeTe for homogeneous crystal nucleation and growth (see article). (c)-(h) instantaneous crystal growth velocities computed as $v_g = dV_c/dtS_{ac}^{-1}$ as described in Ref. 1. The region highlighted in gray in panels c)-h) corresponds to the time interval over which we estimated the average crystal growth velocities reported in Table 2 (Bulk$_{homo}$) in the article. We start computing the instantaneous velocity when all the crystalline nuclei become overcritical.

Table S1. Two-dimensional diffusion coefficient D as a function of time of the confined GeTe slab in GeTe/$TiTe_2$-like superlattice extracted from NVE simulations at the average temperatures given in the first column. We computed D from the two dimensional mean square displacement in the plane perpendicular to the slab thickness in the superlattice as $< x^2 > + < y^2 > = 4Dt$. The diffusion coefficient in the slab is compared to those in the bulk at the experimental density of the amorphous phase from Ref. 2. The values for D in the bulk at the density of the slab which is close to that of crystalline α-GeTe are expected to be lower than those reported in the last column of the table which correspond to the experimental density of the amorphous phase.

Temperature (K)	D (10^{-6}cm^2/s)	
	GeTe/$TiTe_2$-like superlattice	Bulk from Ref. 2
512	0.6	1.5
561	1.2	2.6
614	2.8	4.5
652	4.9	6.2
709	8.2	9.4
750	12.9	12.3

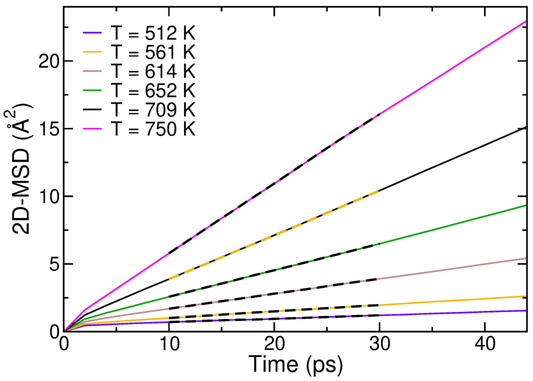

Figure S8. Two-dimensional mean square displacement (MSD) as a function of time from NVE simulations at the average temperatures given in the inset.

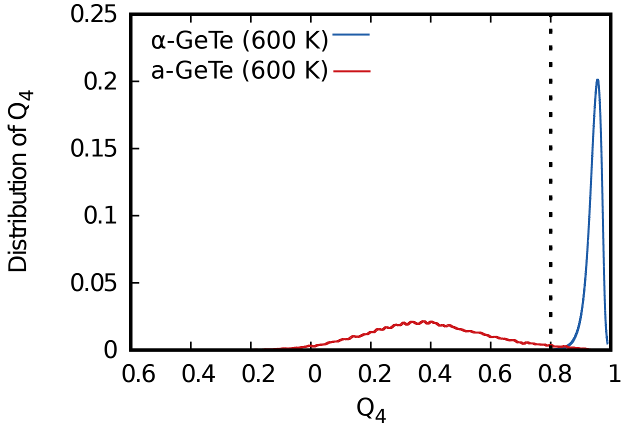

Figure S9. The normalized distribution of the Q_4^{dot} order parameter for amorphous GeTe overheated at 600 K (red curve) and for crystalline α-GeTe at 600 K (blue curve). The vertical dashed line sets the threshold to identify crystalline atoms.

References

1. Xu, Y. *et al.* Unraveling crystallization mechanisms and electronic structure of phase-change materials by large-scale ab initio simulations. *Adv. Mater.* **34**, 2109139, DOI: https://doi.org/10.1002/adma.202109139 (2022).

2. Gabardi, S. *et al.* Atomistic simulations of the crystallization and aging of GeTe nanowires. *J. Phys. Chem C* **121**, 23827–23838, DOI: 10.1021/acs.jpcc.7b09862 (2017).
